# Association of BMI with overall survival in patients with mCRC who received chemotherapy versus EGFR and VEGF-targeted therapies

**DOI:** 10.1002/cam4.490

**Published:** 2015-07-25

**Authors:** Gargi S Patel, Shahid Ullah, Carol Beeke, Paul Hakendorf, Robert Padbury, Timothy J Price, Christos S Karapetis

**Affiliations:** 1 Department of Medical Oncology, Flinders Medical CentreAdelaide, South Australia, Australia; 2 Flinders Centre for Innovation in Cancer, Flinders UniversityAdelaide, South Australia, Australia; 3 Flinders Centre for Epidemiology and Biostatistics, School of Medicine, Flinders UniversityAdelaide, South Australia, Australia; 4 Department of Surgery, Flinders Medical CentreAdelaide, South Australia, Australia; 5 Department of Medical Oncology, The Queen Elizabeth Hospital, AdelaideSouth Australia, Australia; 6 School of Medicine, University of AdelaideAdelaide, South Australia, Australia

**Keywords:** Bevacizumab, BMI, colorectal cancer, metastatic, targeted therapies

## Abstract

Although a raised body mass index (BMI) is associated with increased risk of colorectal cancer (CRC) and recurrence after adjuvant treatment, data in the metastatic setting is limited. We compared overall survival (OS) across BMI groups for metastatic CRC, and specifically examined the effect of BMI within the group of patients treated with targeted therapies (TT). Retrospective data were obtained from the South Australian Registry for mCRC from February 2006 to October 2012. The BMI at first treatment was grouped as underweight <18.5 kg/m^2^, Normal = 18.5 to <25 kg/m^2^, Overweight = 25 to <30 kg/m^2^, Obese I = 30 to <35 kg/m^2^, Obese II ≥35 kg/m^2^. Of 1174 patients, 42 were underweight, 462 overweight, 175 Obese I, and 77 Obese II. The OS was shorter for patients who were underweight and overweight compared to normal (OS 13.7 and 22.3 vs. 24.1 months, respectively, hazard ratio [HR] 2.21 and 1.23). The adjusted median OS was longer for normal versus overweight or obese I patients receiving chemotherapy + targeted therapy (35.7 vs 25.1 or 22.8 months, HR 1.59 and 1.63, respectively) with no difference in OS for chemotherapy alone. On breakdown by type of targeted therapy, overweight and obese I patients had a poorer outcome with Bevacizumab. The BMI is predictive of a poorer outcome for underweight and overweight patients in the whole population. Of those receiving chemotherapy and targeted therapy, BMI is an independent predictor for OS for overweight and obese I patients, specifically for those treated with Bevacizumab. Patients who are overweight or obese (group I) may be a target group for lifestyle and nutrition advice to improve OS with TT.

## Introduction

Overweight and obesity trends are rapidly rising in developed countries such as Australia, Canada, USA, and the UK, with over 50% of the population categorized as overweight/obese in 2004 1. This rate is predicted to rise to over 60% by the end of this decade, leading to a significant comorbidity in the majority of our population. Obesity has been established as a prognostic factor for the development of colorectal cancer (CRC), accounting for up to 35% of CRC cases, and is associated with a higher risk of recurrence and colon cancer-specific mortality in the adjuvant setting 2–6.

Preclinical work suggests a link between insulin resistance, related to obesity, and colorectal carcinogenesis 6. Factors that are raised in obesity, such as blood insulin levels, insulin-like growth factor (IGF) 1, leptin, interleukin 6, and tumor necrosis factor-alpha (TNF-*α*) are associated with activation of intracellular signaling pathways, such as the PI3kinase or mitogen-activated protein kinases (MAPK) pathway 7,8. These pathways are also targeted by epidermal growth factor inhibitors (EGFR), for example, cetuximab and crosstalk with the above-mentioned proinflammatory pathways may lead to resistance to EGFR inhibitors 9. Furthermore, elevated leptin levels are correlated with production of vascular endothelial growth factor (VEGF) 10, leading to angiogenesis, which may lead to resistance to VEGF-targeted drugs, such as bevacizumab in obesity-associated cases. From this data, we hypothesized that adiposity, and a raised BMI, may be negatively associated with survival in patients with CRC, especially those treated with targeted therapies (TT) such as bevacizumab and cetuximab, versus those receiving chemotherapy alone.

Few studies have demonstrated evidence pertaining to the effect of obesity on clinical outcome in patients with metastatic colorectal cancer (mCRC), and results are conflicting. Two retrospective trials have reported on the association of body mass index (BMI) with overall survival (OS) and/or time to progression (TTP) in patients treated with bevacizumab 11,12. One study found an increase in BMI to be associated with a better OS, for those patients treated with chemotherapy, but no significant association in a group treated with chemotherapy and bevacizumab 11. However, Guiu *et al*. showed that high BMI and visceral fat area (VFA) were associated with reduced response rates, disease-free survival and OS in patients treated with bevacizumab and chemotherapy, but there was no significant association between these factors and outcome in patients treated with chemotherapy 12. The CO.17 study examined the association between various factors, including BMI, comorbidity and age, and OS in cetuximab-treated patients with mCRC and found no difference in OS according to BMI 13. On the other hand, studies have reported either no association between BMI and OS, in patients with mCRC undergoing hepatectomy 14, or a negative effect only in men with CRC 15. Further work is necessary in this field to elucidate the effect, if any, of BMI upon outcome for mCRC patients. Our aim was to compare OS across BMI groups for mCRC, and specifically examine the effect within the group of patients treated with TT. In particular, the primary objective of the study was to examine whether BMI was prognostic of OS, defined as the interval from the first diagnosis of mCRC to death or last follow up. The secondary objective was to examine the association of BMI with OS for the subgroup of patients receiving therapies targeted to the EGFR or VEGF, according to the biological rationale discussed above.

## Materials and Methods

Retrospective data were collected from the South Australian Clinical Registry for mCRC 16 which is a state-wide population-based database including data from all patients diagnosed with mCRC from February 1, 2006. Data on patient details such as age, sex, demographics, tumor characteristics, including site, histopathological subtype, differentiation, and metastatic site, investigations, treatment, such as surgical procedures, chemotherapy, targeted therapy, radiotherapy, radiofrequency ablation and selective internal radiation therapy and outcomes are included. Patient selection is based upon the relevant International Classification of Diseases codes from inpatient and outpatient encounters, histopathology reports, clinical notification, attendance at multidisciplinary meetings and death audits. Cancer-specific mortality was obtained for each patient through medical record review and electronic linkage with the State death records.

Baseline BMI was calculated as weight in kilograms divided by height, in metres squared, as recorded at first diagnosis of mCRC, prior to treatment with chemotherapy. BMI groups for analysis were based upon the World Health Organisation (WHO) criteria 17 as below:

Underweight = <18.5 kg/m^2^, normal weight = 18.5 to <25 kg/m^2^, Overweight = 25 to <30 kg/m^2^, Obese I = 30 to <35 kg/m^2^, Obese II/III = 35 kg/m^2^ or greater.

Overall survival analysis was initially carried out for the whole population, and then within subgroups according to treatment type. Patients treated with chemotherapy alone were classified as the chemotherapy group and patients who received targeted treatments +/− chemotherapy at any point in the treatment for metastatic disease were classed as the chemotherapy and antibody treatment group. The underweight group was excluded from analysis by treatment type due to the extremely small numbers in the chemotherapy and antibody treatment group, which did not allow for multivariate regression analysis for OS. Data regarding progression free survival or change in BMI over the course of treatment was not robustly available for all patients and therefore was not analyzed.

All analyses were performed using STATA software (Version 11: Stata Corp., TX).

Cox proportional hazards models were fitted to evaluate the cumulative survival probabilities among the BMI groups. The time to event was defined as the interval from the first diagnosis of mCRC to death or last follow-up, which comes first. The census date was 31 October 2012.

Multivariate Cox PH regression analysis was used to adjust for confounders such as gender, age, number of metastatic sites, metachronous versus synchronous presentation (whether patients presented with stage I–III disease and subsequently developed metastases, versus metastatic disease at presentation), number of lines of chemotherapy, and the number of lines of TT received. A PH assumption was tested and confirmed to satisfy assumption. OS were evaluated by Kaplan–Meier survival curves and groups were compared by log-rank test. Analysis of variance (ANOVA) or chi-squared tests, including post hoc corrections where necessary, were used to compare patient demographic groups. Although the number of patients is unequal for BMI groups, this lack of balance does not present serious problems for one factor ANOVA 18,19.

## Results

### Patient demographics

In total, 1174 patients were available for the calculation of BMI at first presentation of mCRC. BMI was only recorded on the database for patients who received chemotherapy. A further 285 patients were treated with chemotherapy but BMI records were not available in the notes.

Of the 1174 patients included in the study, 42 (3.6%) patients were underweight, 418 (35.6%) were of normal weight, 462 (39.4%), overweight, 175 (14.9%), obese (group 1) and 77 (6.6%), obese (groups 2 and 3) with a BMI of over 35 kg/m^2^. Patient demographics for the all patients are displayed in Table1. There were no significant differences between BMI groups for the whole dataset in terms of age, number of metastatic sites, number of lines of chemotherapy or antibody therapy or by KRAS mutation status. However, within the whole group, patients who were underweight were more likely to be female rather than male. A higher proportion of normal weight patients presented with synchronous metastasis (metastatic disease at first presentation) compared to the underweight, overweight or obese patient groups. Normal weight patients were more likely to receive chemotherapy alone compared to overweight patients who were more likely to receive chemotherapy and at least one line of targeted therapy (75.1% of normal weight patients received no lines of antibody treatment compared to 64.5% of overweight patients). No significant differences were detected between the obese patient groups compared to the normal under- or overweight groups.

**Table 1:** Patient and tumor baseline characteristics for all patients

Characteristics	BMI (kg/m^2^)					*P*-value
	Underweight (*n* = 42)	Normal (*n* = 418)	Overweight (*n* = 462)	Obese I (*n* = 175)	Obese II (*n* = 77)	
Age (mean)	65.4	64.6	64.4	62.9	63.3	0.34
Sex (%)						
Female	59.5	37.7	32.9^1^	39.7	46.2	<0.01
Male	40.5^1^	62.3	67.1^1^	60.3	53.8	
Group (%)						
Chemotherapy only	81.0	75.1^1^	64.5^1^	66.3	67.9	<0.01
Antibody tx	19.0	24.9^1^	35.5^1^	33.7	32.1	
Stage at dx (%)						
Synchronous disease	61.9	77.8^1^	64.3	60.6	57.7	<0.001
No. of met sites						
1	76.2	73.9	74.0	73.7	71.8	0.98
>1	23.8	26.1	26.0	26.3	28.2	
Chemo line (%)						
1	50.0	45.5	39.9	45.1	52.6	0.55
2	31.0	30.1	30.8	31.4	23.1	
3	16.7	16.3	18.4	16.0	14.1	
4	2.4	6.2	7.2	6.3	9.0	
5+	–	1.9	3.7	1.1	1.3	
Ab line (%)						
0	81.0	75.1^1^	64.5^1^	66.3	67.9	0.05
1	16.7	17.5^1^	27.9^1^	25.7	25.6	
2	–	5.7	5.4	6.3	6.4	
3	2.4	1.7	2.2	1.7	–	
KRAS mutation	4.8	10.8	12.3	12	7.7	0.93

Underweight, BMI < 18.5, Normal, BMI 18.5–24.9; Overweight, BMI 25.0–29.0; Obese I, BMI 30.0–34.9; Obese II, BMI ≥ 35; Percent of patients in each group by BMI category are shown. *P*-values are based on ANOVA for continuous data and Chi-square test for categories.

1 Denotes a subset of BMI categories whose column proportions differ significantly from the other columns at the 0.5 level.

Of all the patients analyzed above, 814 received chemotherapy alone compared to 360 patients who received chemotherapy and at least one line of targeted therapy, including bevacizumab, cetuximab, panitumumab, and/or regorafenib. Patient demographics for these two groups of patients, split by BMI are shown in Table2, with the only significant difference in both groups being the stage of disease at presentation. Within both treatment groups, normal weight patients were more likely to present with synchronous disease compared to overweight or obese patients, as described for the whole patient group above. As only 8 patients in the underweight group received chemotherapy and antibody treatment, we could not carry out a multivariate analysis for this subgroup, and thus the underweight patients were excluded from this part of the study.

**Table 2:** Patient and tumor baseline characteristics for patients receiving chemotherapy alone or chemotherapy and targeted therapies

Characteristics	Chemotherapy only					*P*-value	Chemotherapy and antibody treatment					*P*-value
	BMI (kg/m^2^)						BMI (kg/m^2^)					
	Under-weight (*n* = 34)	Normal (*n* = 314)	Over-weight (*n* = 298)	Obese I (*n* = 116)	Obese II (*n* = 52)		Under-weight (*n* = 8)	Normal (*n* = 104)	Over-weight (*n* = 164)	Obese I (*n* = 59)	Obese II (*n* = 25)	
Age (mean)	66.0	65.9	65.8	64.4	63.5	0.36	62.5	60.9	61.9	59.9	63.1	0.57
Sex (%)												
Female	58.8^1^	36.1	33.2^1^	44.0	47.2	0.01	62.5	42.7	32.3	31.0	44.0	0.16
Male	41.2^1^	63.9	66.8^1^	56.0	52.8		37.5	57.3	67.7	69.0	56.0	
Synchronous disease (%)	61.8	77.7^1^	65.9	56.0	58.5	0.001	62.5	77.9^1^	61.6	69.5	56.0	0.05
No. of met sites (%)												
1	73.5	73.2	75.5	73.3	71.7	0.96	87.5	76.0	71.3	74.6	72.0	0.81
>1	26.5	26.8	24.5	26.7	28.3		12.5	24.0	28.7	25.4	28.0	
No of lines of chemo (%)												
1	55.9	51.3	45.1	54.3	66.0	0.38	25.0	27.9	30.5	27.1	24.0	0.74
2	32.4	31.2	35.4	30.2	18.9		25.0	26.9	22.6	33.9	32.0	
3	8.8	12.4	14.1	11.2	7.5		50.0	27.9	26.2	25.4	28.0	
4	2.9	3.2	4.0	4.3	7.5			15.4	12.8	10.2	12.0	
5+	–	1.9	1.3	–	–		–	1.9	7.9	3.4	4.0	
No. of lines of antibody (%)												
0	_	_	_	_	_		_	_	_	_	–	0.56
1	–	–	–	–	–		87.5	70.2	78.7	76.3	80.0	
2	_	_	_	_	_			23.1	15.2	18.6	20.0	
3	–	–	–	–	–		12.5	6.7	6.1	5.1	–	
%pt KRAS tested	5.9	22.9	20.8	20.7	11.3	0.08	75.0	45.2	49.4	49.2	60.0	0.41
Type of KRAS (%)												
KRAS wild type	50.0	55.6	41.9	58.3	50.0	0.53	83.3	72.3	74.1	62.1	80.0	0.65
KRAS mutant	50.0	44.4	58.1	41.7	50.0		16.7	27.7	25.9	37.9	20.0	

*P*-values are based on ANOVA for continuous data and Chi-square test for categories.

1 Denotes a subset of BMI categories whose column proportions differ significantly from the other columns at the 0.5 level.

### Types of TT used

The majority of patients received bevacizumab, either in the first or second line of treatment, with the mode falling in the group of patients receiving second line treatment, as shown in Table3. Significantly fewer patients received EGFR-TT, with the mode falling in the 4th line of treatment for both cetuximab and panitumumab. Within this group, cetuximab was the preferred option. Very few patients received regorafenib, usually within the context of a clinical study.

**Table 3:** Number of patients receiving targeted agents by line of treatment

	1 st line	2nd line	3rd line	4th line	5th line and beyond
Bevacizumab	91	**126**	43	21	19
Cetuximab	5	21	30	41	32
Panitumumab	4	3	7	10	3
Regorafenib	1	0	4	0	1
None	251	202	268	280	297

Numbers in bold denote the mode for each group.

### OS analysis

**Figure 1:**
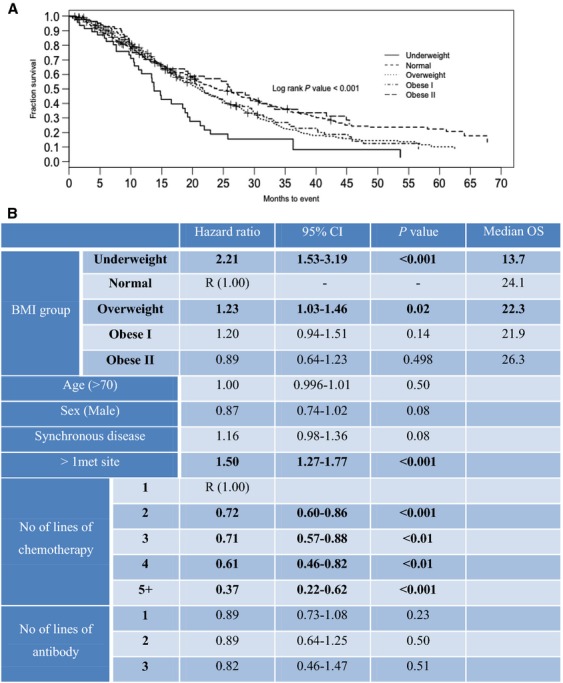
Kaplan–Meier survival curves (A) and multivariate Cox proportional hazard model (hazard ratio [HR] and and 95% CI) for overall survival by BMI for the whole population (B). Model was adjusted by potential confounders: age, sex, synchronous disease, >1 met site, number of lines of chemotherapy and number of lines of antibody.

Multivariate analysis by cox regression modeling was carried out for the whole dataset and adjusted for age, sex, synchronous disease at presentation, number of metastatic sites, number of lines of chemotherapy, and number of lines of targeted treatment. This demonstrated a significant reduction in OS for patients in the underweight group of 13.7 months compared to 24.1 months for the normal weight group, with a hazard ratio (HR) of 2.21 (95% CI 1.53–3.19; *P* < 0.001) (Fig.1B). Furthermore, there was a significant increase in the risk of death within the overweight group, HR 1.23(95% CI 1.03–1.46, *P* = 0.02), with no significant differences in survival for the obese groups compared to the normal weight group. Independent predictors of survival included greater than one metastatic site (HR 1.50, 95% CI 1.27–1.77 *P* < 0.001) and number of lines of chemotherapy treatment, with the HRs reducing in proportion to the number of lines of chemotherapy tolerated (Fig.1B). However, the HRs for number of lines of antibody used were not statistically significant.

**Figure 2:**
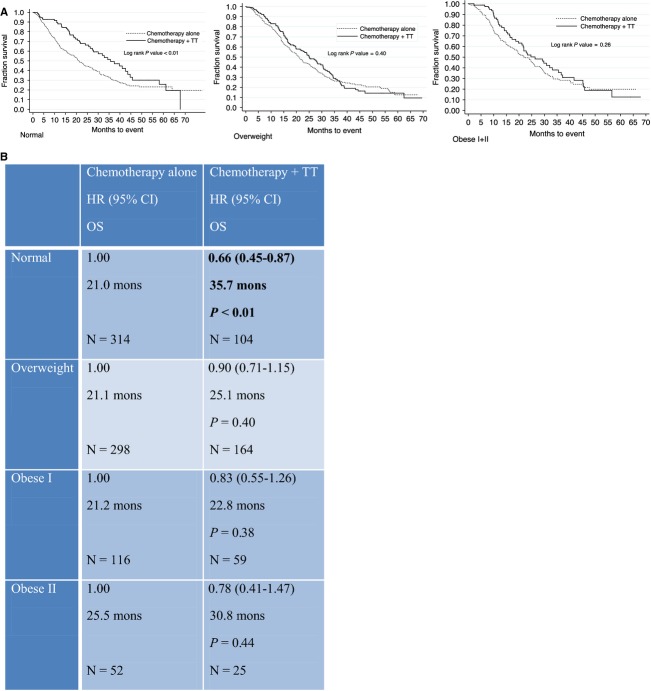
Kaplan–Meier survival curves (A), and Cox proportional hazard model (hazard ratio [HR], 95% CI and median overall survival [OS]) for overall survival for patients receiving chemotherapy alone versus chemotherapy and TT (B).

Overall survival analysis for patients treated with chemotherapy alone demonstrated no difference in median OS by BMI. The underweight group was not included in this analysis due to the small numbers within subgroups, which did not allow for multivariate analysis. HRs for OS in this group were 1.08, 1.07, and 0.84 for the overweight, obese I, and obese II/III groups, respectively, when compared with the normal group, with no significant *P* values (Table S1). Within this analysis, age, number of metastatic sites, and number of lines of chemotherapy were independent predictors for survival, as expected. Comparison of median OS for patients receiving chemotherapy alone versus chemotherapy and TT, within each BMI group demonstrated a survival benefit for the addition of TT in the normal group only (OS 21.0 vs. 35.7 months, HR 0.66, *P*-value < 0.01) (Fig.2).

Within the prespecified patient group receiving TT and chemotherapy, median OS was significantly longer for normal weight patients compared to overweight and obese I patients (35.7 months, vs. 25.1 and 22.8 months respectively), with HR of 1.59, *P* = 0.006, 1.63, and 0.049, respectively, as shown in Table4. Upon breakdown by type of TT, this difference was significant for patients receiving VEGF-targeted treatment but not for EGFR TTs, likely due to the small numbers in the latter group. Patients receiving both VEGF and EGFR-TT were excluded from the analysis presented in Table4. Median OS for overweight and obese I patients was half than that seen for normal weight patients for those treated with VEGF-TT (17.5 months, and 16 months compared to 36.1 months, respectively, with HR of 2.08, *P* = 0.001, 2.67, 0.004).

**Table 4:** Multivariate Cox proportional hazard model (Hazard ratios [HR], 95% CI and overall survival [OS]) for BMI groups by type of targeted therapy

	Normal	Overweight	Obese I	Obese II
	HR (95% CI)	HR (95% CI)	HR (95% CI)	HR (95% CI)
	OS	OS	OS	OS
A11TT (*N* = 352)	1.00	**1.59 (1.14–2.21)**	**1.63 (1.01–2.65)**	1.11(0.60–2.06)
	**35.7 months**	**25.1 months**	**22.8 months**	30.8 months
		***P***** = 0.006**	***P***** = 0.049**	*P* = 0.740
	*N* = 104	*N* = 164	*N* = 59	*N* = 25
VEGFR TT (*N* = 200)	1.00	**2.08 (1.35–3.21)**	**2.67 (1.37–5.20)**	0.81 (0.30–2.21)
	**36.1 months**	**17.5 months**	**16.0 months**	63.5 months
		***P***** = 0.001**	***P***** = 0.004**	*P* = 0.677
	*N* = 63	*N* = 91	*N* = 35	*N* = 11
EGFR TT (*N* = 106)	1.00	1.33 (0.72–2.47)	0.95 (0.39–2.27)	1.90 (0.67–5.38)
	**40.8 months**	30.3 months	41.6 months	31.8 months
		*P* = 0.356	*P* = 0.900	*P* = 0.230
	*N* = 27	*N* = 52	*N* = 17	*N* = 10

**Figure 3:**
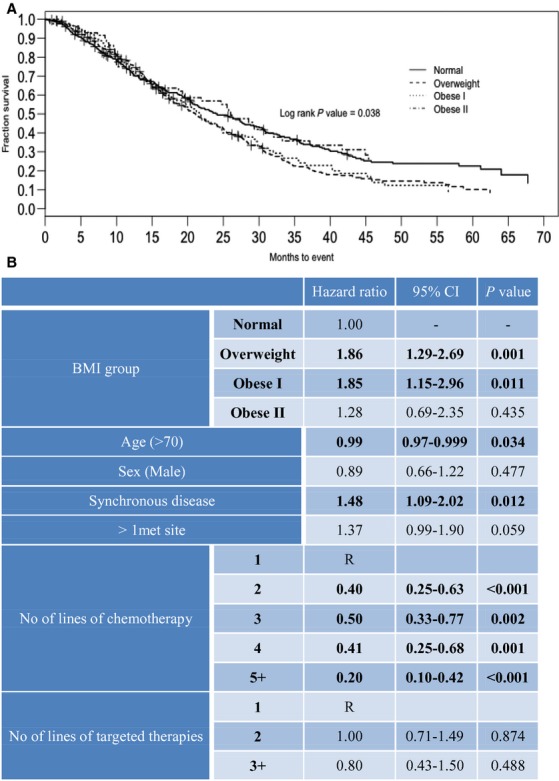
Kaplan–Meier overall survival curves (A) and multivariate Cox proportional hazard model (Hazard ratio [HR] and and 95% CI) for overall survival by BMI for patients receiving any TT (B). Model was adjusted by potential confounders: age, sex, synchronous disease, >1 met site, number of lines of chemotherapy and number of lines of TT.

The results described for the group of patients receiving any TT retained significance on adjustment for age, sex, synchronous disease, number of metastatic sites, number of lines of chemotherapy, and TT, by multivariate analysis (Fig.3B). Independent predictors of death included age of greater than 70, and synchronous disease at presentation. The number of lines of chemotherapy received, but not the number of lines of TT, was an independent predictor for survival. The Kaplan–Meier survival curves corresponding to this data are shown in Figure3A.

The interactions of age with BMI and sex with BMI were not significant and were not included in the model of VEGFR TT, EGFR TT, and all TT for evaluating the cumulative survival probabilities among the BMI groups.

## Discussion

Upon analysis of the entire patient group, irrespective of treatment, a poor OS for underweight patients was demonstrated with a median OS of 13.7 months compared to 24.1 months, and a HR of 2.21. Patients in the overweight group also demonstrated a poorer OS compared to the normal group with a median OS of 22.3 compared to 24.1 months, with a HR of 1.23. No significant difference was demonstrated between the normal and obese groups. These differences in OS are likely to be due to different pathologies in the two different BMI groups. An underweight BMI at presentation may in fact represent cachexia associated with aggressive metastatic disease, which may be related to a poor performance status, thus preventing effective chemotherapeutic treatment. Indeed patients within this group were the most likely to receive chemotherapy alone compared to chemotherapy plus TT, as demonstrated in Table1. This finding is supported by a study carried out by Parsons et al. who investigated the association of body composition parameters and outcome for patients receiving hepatic arterial infusion chemotherapy for metastatic liver disease 20. Sarcopenic patients demonstrated a poor OS of 103 days versus 312 days, but their results were not significant in a small patient population of 20 sarcopenic patients. Van Vledder et al. demonstrated that sarcopenia was an independent predictor of worse-recurrence free survival and OS after surgery for colorectal liver metastases (HR of 1.88, *P* < 0.01, 2.53, 0.001, respectively) 21.

Conversely, overweight patients were also found to have a statistically significant reduction in OS compared to normal weight patients. However, this group was more likely to demonstrate good prognostic factors, such as metachronous disease at presentation and treatment with at least one line of TT compared to normal weight patients. The reasons behind these discrepancies is not entirely clear but may be related. For instance, if overweight patients had previously received adjuvant chemotherapy, they may be more likely to be offered antibody therapy in conjunction with chemotherapy compared to chemotherapy-naive patients. The overweight group would be expected to do as well as, if not better than the normal weight group in terms of OS, but this was not the case. We conclude that an overweight BMI may represent an independent, poor, prognostic indicator for survival in patients undergoing chemotherapy +/− TT for metastatic CRC.

On analysis by treatment type, the most significant finding is that BMI is an independent predictor for poorer outcome for patients in the overweight and obese I group for patients treated with chemotherapy and TT but not for patients treated with chemotherapy alone. The only significant difference in patient demographics by BMI for this group of patients was that normal weight patients were more likely to present with synchronous metastases, which has been shown to be associated with a poorer OS, compared to the overweight or obese groups. Although there is evidence that male gender is associated with an earlier occurrence of CRC 22, our interaction analysis discovered no effect for sex by BMI in this population. However, the numbers of patients treated with EGFR TT were small in this study.

Analysis by type of TT used demonstrates a worse median OS for patients in the overweight and obese I group compared to the normal group for patients treated with VEGF inhibitors (i.e., Bevacizumab) but not with EGFR TT (Table4). No significant differences were noted between OS for the obese II/III group and the normal group, which may be related to the small numbers within this group (*n* = 25). These findings support the predetermined biological hypothesis proposing resistance to anti-angiogenic therapy due to the potential inflammatory and proangiogenic properties associated with adipose tissue. Interestingly, although adiposity is associated with IGFR activation, a proposed mechanism of resistance to EGFR TT, no difference in OS was found in between the BMI groups for patients treated with EGFR TT alone. However, total numbers within the EGFR TT group were small within our study.

Our results are in concordance with Guiu et al. who retrospectively demonstrated that a high VFA higher than the median is associated with a poorer response to VEGF-TT as well as shorter OS in patients treated with bevacizumab and chemotherapy in the first line treatment of metastatic CRC 12. However, in their study, multivariate analysis did not confirm that a BMI of greater than 23.6 (median in their population) was predictive of response or prognostic of OS. Faruk Aykan et al. also report a similar effect, with a reduction in progression-free survival for patients with mCRC, treated with bevacizumab in a small (80 patients) retrospective series 23. They used a binary cut off of 25 for the BMI in this study. We demonstrate, for the first time, using the WHO specified BMI groups, that an overweight and obese (I) BMI is prognostic of poor OS and may be predictive of a lack of response to VEGF-TT within a large multicenter database.

In contrast to our results, Simkens et al. demonstrate that BMI is an independent prognostic indicator of survival for patients receiving chemotherapy but not for those receiving chemotherapy and bevacizumab 11. However, the overweight and obese patients included in their studies demonstrated a better performance status than those in the normal weight group, as their patient population was derived from a clinical trial database with a performance status of less than or equal to 2 as an inclusion criteria. The authors comment that this discrepancy may well have affected the results presented. However, our database is more likely to be more representative of the general population as it is inclusive of patients both on clinical trials and off study. One other retrospective study has demonstrated no association between BMI and either TTP or OS for mCRC patients treated with bevacizumab, but this was a small study of only 184 patients from a single center 24.

One major limitation of our study is that it was retrospective in nature, as are the other studies examining the association of BMI and outcome for patients with mCRC in the literature 11,12. Furthermore, our results do not account for a potential variable distribution of VEGF-TT in adipose tissue, which may account for the reduced efficacy of these drugs in the overweight population. One hypothesis for poorer outcomes in overweight or obese patients relates to dose capping of chemotherapy in these individuals 25,26 which may be an important confounding factor within our study. Although we could not obtain exact chemotherapy doses for all patients, we have tried to control for this factor by comparing OS by BMI within the group of patients treated with chemotherapy alone. There are no significant differences in OS between normal, overweight, and obese patients treated with chemotherapy alone (21, 21.1, and 22 months, respectively, Fig.2, panel B). Therefore, we have concluded that inadequate chemotherapy dosing is unlikely to be a confounding factor for our results.

Prospective studies are now required to examine the pharmacokinetics and pharmacodynamics of such TT in overweight patients, as well as to gather data on cardiovascular comorbidities, smoking, and drug histories, in order to validate these results.

In conclusion, we found that BMI is an independent prognostic indicator of a poor OS for patients who are overweight and obese I, specifically for patients receiving chemotherapy and VEGF-TT. Patients within these groups may represent a target group for lifestyle and nutritional advice in order to improve OS with TT. Feasibility studies have demonstrated an improvement in health-related quality of life, symptoms, dietary habits, and BMI in CRC patients in the adjuvant setting with a relatively low cost technique, such as a telephone-delivered health behavior intervention 27,28. Further studies are required to investigate if such interventions are effective, both at reducing BMI and improving outcome for patients with mCRC.

## Supporting Information

**Table S1.** Multivariate Cox proportional hazard model (hazard ratio [HR] and 95% CI) for overall survival by BMI for chemotherapy only group, model was adjusted by synchronous disease, >1 met site, number of lines of chemotherapy and number of lines
