# Supplementary material for: Association of BMI with overall survival in patients with mCRC who received chemotherapy versus EGFR and VEGF-targeted therapies
Source: Cancer Med. 2015 Jul 25;4(10):1461–71. doi: 10.1002/cam4.490 (PMC4618617; doi:10.1002/cam4.490)
Supplement: Supplementary file 1 [file cam40004-1461-sd1.pdf]

Supplementary Figure

		Hazard ratio	95% CI	P value
BMI group	**Normal**	1.00	-	-
	**Overweight**	1.08	0.88-1.33	0.454
	**Obese I**	1.07	0.81-1.42	0.629
	**Obese II**	0.84	0.57-1.23	0.375
Age (>70)		**1.01**	**1.00-1.02**	**0.033**
Sex (Male)		0.85	0.71-1.03	0.091
Synchronous disease		1.14	0.93-1.39	0.201
> 1met site		**1.54**	**1.26-1.88**	**<0.001**
No of lines of chemotherapy	**1**	R		
	**2**	0.82	0.66-1.00	0.055
	**3**	**0.72**	**0.55-0.95**	**0.018**
	**4**	**0.62**	**0.40-0.85**	**0.027**
	**5+**	**0.38**	**0.16-0.93**	**0.034**

Supplementary Table 1. Multivariate Cox proportional hazard model (hazard ratio (HR) and 95% CI) for overall survival by BMI for chemotherapy only group, Model was adjusted by synchronous disease, >lmet site, number of lines of chemotherapy and number of lines
